# Propensity score-based comparison of high-risk coronary artery bypass grafting vs. left ventricular assist device implantation in patients with coronary artery disease and advanced heart failure

**DOI:** 10.3389/fcvm.2024.1430560

**Published:** 2024-10-01

**Authors:** Gaik Nersesian, Alaa Abd El Al, Felix Schoenrath, Armin Zittermann, Laurenz Hell, Volkmar Falk, Theo M. M. H. de By, Henrik Fox, Rene Schramm, Michiel Morshuis, Jan Gummert, Evgenij Potapov, Sebastian V. Rojas

**Affiliations:** ^1^ Department of Cardiothoracic and Vascular Surgery, Deutsches Herzzentrum der Charité (DHZC), Berlin, Germany; ^2^ DZHK (German Centre for Cardiovascular Research), Partner Site Berlin, Berlin, Germany; ^3^ Department of Cardiovascular Surgery, Charité – Universitätsmedizin Berlin, Berlin, Germany; ^4^ Clinic for Thoracic and Cardiovascular Surgery, Herz- und Diabeteszentrum NRW, Ruhr-University Bochum, Bad Oeynhausen, Germany; ^5^ Department of Health Sciences and Technology, ETH Zurich, Zürich, Switzerland; ^6^ Berlin Institute of Health, Berlin, Germany; ^7^ Department of Health Sciences and Technology, Institute of Translational Medicine, Swiss Federal Institute of Technology, Translational Cardiovascular Technologies, Zurich, Switzerland; ^8^ EUROMACS Registry, EACTS, Windsor, United Kingdom

**Keywords:** LVAD, heart failure, CABG, coronary artery disease, coronary revascularization

## Abstract

**Objectives:**

Revascularization in patients with severely reduced left ventricular function and coronary artery disease (CAD) is associated with a high perioperative risk. In this setting, implantation of a durable left ventricular assist device (LVAD) might be an alternative.

**Methods:**

We retrospectively compared the outcomes of adult patients with CAD and a left ventricular ejection fraction (LVEF) ≤ 25% who underwent coronary artery bypass grafting (CABG) vs. LVAD implantation. Propensity score (PS) matching was performed for statistical analysis, resulting in 168 pairs.

**Results:**

In the PS-matched cohorts, the mean age was 62 years; one third had a history of myocardial infarction, 11% were resuscitated, half of the patients were on inotropic support, and 20% received preoperative mechanical circulatory support. LVAD patients required significantly longer ventilation (58 h [21, 256] vs. 16 h [9, 73], *p* < 0.001) and had a longer ICU stay (11d [7, 24] vs. 4d [2, 10], *p* ≤ 0.001) compared to CABG patients The incidence of postoperative renal replacement therapy (2 [1.2%] vs.15 [8.9%], *p* = 0.002) and temporary mechanical circulatory support was lower in the LVAD group (1 [0.6%] vs. 51 [30.4%], *p* ≤ 0.001). The in-hospital stroke rate was similar (LVAD 7 [5.4%] vs. CABG 8 [6.2%], *p* = 0.9). In-hospital survival, 1-year survival, and 3-year survival were 90.5% vs. 85.5% (*p* = 0.18), 77.4% vs. 68.9% (*p* = 0.10) and 69.6% vs. 45.9% (*p* < 0.001), for CABG and LVAD patients respectively.

**Conclusion:**

Patients with CAD and advanced HF demonstrate better mid-term survival if they undergo CABG rather than LVAD implantation.

## 1 Introduction

Coronary artery disease (CAD) and acute myocardial infarction (AMI) are the primary contributors to morbidity and mortality in high-income countries (1, 2). CAD leads to acute and/or chronic myocardial ischemia, which causes fibrotic remodeling of the myocardium and loss of contractility, and can result in advanced heart failure (HF) (2). There is an ongoing debate as to the most appropriate approach in CAD patients with advanced HF—optimal medical therapy and device treatment (OMDT) or myocardial revascularization with either endovascular or surgical approach (3).

While it is certain that future ischemic events can be prevented with coronary artery bypass grafting (CABG), it can be difficult to determine whether the myocardium has the potential for reverse remodeling after revascularization. In patients with end-stage HF, and ischemic heart disease implantation of a durable left ventricular assist device (LVAD) may also be considered to improve cardiac output and relieve symptoms (3).

The existing literature demonstrates that CABG not only enhances survival in patients with CAD and advanced HF compared to OMDT alone but also protects against potential future AMI (4, 5). However, most studies to date have primarily examined patients with a left ventricular ejection fraction (LVEF) of 30%–35%, a setting in which durable mechanical circulatory support (MCS) is rarely utilized. In our analysis we focused specifically on CAD patients with an LVEF of 25% and lower and symptoms of advanced heart failure (3). In this subgroup of patients, CABG is associated with an increased perioperative risk, making LVAD implantation a potential alternative (3, 6, 7).

## 2 Materials and methods

### 2.1 Patient population

Adult cardiac patients from two tertiary cardiac centers who underwent either CABG or LVAD implantation and met the inclusion criteria were retrospectively enrolled in the analysis.

Inclusion criteria

• Adult cardiac patients
• CAD with LVEF ≤ 25%
• No acute myocardial infarction within 7 days before surgery
• CABG or LVAD implantation between 01/2011–12/2022.

Exclusion criteria

• Concomitant procedures during CABG or LVAD implantation (e.g., valve replacement, reconstruction, CABG during LVAD implantation)
• LVAD model other than HeartWare HVAD (HW; Medtronic, Minneapolis, MN, USA) or HeartMate 3 (HM3; Abbott, Chicago, IL, USA)
• In order to preclude data duplication and an impact of the surgical technique, we excluded patients who had undergone previous cardiac operations from the analysis.

### 2.2 Ethical statement

The ethics committee of Charité—Universitätsmedizin Berlin approved the study (ethics vote no. EA4/192/22).

### 2.3 Therapy decision making

The surgical strategy was determined by a multidisciplinary heart team, which made the decision to perform either a CABG or LVAD implantation in patients with CAD and severely reduced LVEF based on preoperative diagnostics and co-morbidities. The coronary angiograms were first evaluated by interventional cardiologists and heart surgeons. Based on the coronary anatomy, targets, and overall vessel status, they discussed whether adequate revascularization could be achieved. In patients with poor targets, particularly those with CTO of the LAD without collaterals or severe MR, further evaluation for durable LVAD implantation was conducted.

Additional diagnostics aimed at assessing the severity of heart failure were performed, including but not limited to stress echocardiography with strain measurement and cardiac MRI in patients suspected of having extensive myocardial fibrosis and/or severely reduced contractile reserve.

The findings were discussed by the multidisciplinary heart team, and a patient-specific treatment strategy was established.

### 2.4 Surgical technique

CABG in patients was performed using a conventional median sternotomy approach in both centers. The strategy for circulatory support during the operation included three different approaches:

• Off-pump beating-heart technique
• On-pump beating-heart technique: CPB was utilized, but cardioplegic heart arrest was not induced.
• On-pump arrested-heart technique: CPB was employed, and cardioplegia was administered to arrest the heart.

The circulatory support strategy (if needed during or post CABG) encompassed both CPB and temporary mechanical circulatory support [such as va-ECLS or micro axial transaortic pump (Impella®, Abiomed, Danvers, MA, USA)]. The decision regarding the surgical strategy was made collaboratively by the operating surgeon and the anesthesiologists.

Left ventricular assist device implantation was carried out either via conventional median sternotomy or a minimally invasive approach with a left lateral thoracotomy or bilateral thoracotomy. For LVAD implantation no cardioplegia was administrated.

### 2.5 Endpoints and data assessment

The primary endpoint was all-cause mortality up to a maximum follow-up of 3 years.

Secondary clinical endpoints were stroke, renal replacement therapy (RRT), and need for postoperative temporary MCS (in the CABG group due to post-cardiotomy cardiogenic shock; in the case of LVAD patients temporary right ventricular support). RRT and temporary MCS were assessed until discharge, whereas stroke was assessed during the follow-up.

### 2.6 Statistical analysis

The statistical plan was approved by all authors prior to analysis. Due to the non-randomized group assignment, we generated a propensity score (PS) for each patient. To generate the PS, we used the multivariable logistic regression model with type of treatment (CABG or LVAD) as the binary dependent variable. The model comprised the following baseline covariates: age, sex, body mass index, diabetes mellitus, insulin-dependent diabetes mellitus, major myocardial infarction, mitral regurgitation, cardiac arrest, renal replacement therapy, chronic obstructive pulmonary disease, peripheral vascular disease, stroke, mechanical ventilation, inotropic support, mechanical circulatory support, and the creatinine- and bilirubin-based model for end-stage liver disease excluding INR (MELD-XI) score (calculated using the following formula: 5.11 × ln(bilirubin) + 11.79 × ln(creatinine) + 9.44) (8). All these variables were included regardless of their statistical significance. Matching was performed using a 1:1 ratio with the logit transformed PS. For this, an optimal-matching algorithm with a caliper width of 0.1 standard deviations (SDs) from the linear predictor was used. The balance of risk factors was judged by standardized mean differences (SMDs). The balance is considered satisfactory when the SMD is less than 10% (9). Moreover, we generated Kaplan–Meier estimates for each study group for the primary endpoint up to a maximum follow-up of 3 years. Results were compared using the log-rank test. We also used Cox proportional hazards models, stratified for the matched pairs, for data analysis. Results are presented as hazard ratios (HRs, considering the total number of events and the timing of each event) and 95% confidence intervals (CIs). The proportionality of hazard assumption was satisfied by an evaluation of time-dependent variables, which were the cross products of the predictor variables with event-free outcomes (all *p*-values > 0.05). The follow-up was assessed using the Kaplan–Meier estimator of potential follow-up (10).

To calculate SMD, all continuous parameters in Table 1 are presented as mean with SD. Since all perioperative continuous parameters were non-normally distributed, as assessed by the Kolmogorov-Smirnov test (*p* < 0.05), we present these data as median with 25th and 75th percentiles. The Mann-Whitney *U*-test was applied to compare these parameters for each study group. All categorical variables are summarized as percentages and number of observations. Categorical clinical outcome data are also presented as risk ratios (RRs, total number of events by the end of follow-up) and 95% confidence intervals (CIs) with the LVAD cohort as the reference group. Fisher's exact test was used to compare perioperative outcomes such as the need for hemofiltration and in-hospital mortality. P-values <0.05 were considered statistically significant. We used the statistical software package IBM SPSS, version 24 (IBM Corp, Armonk, New York, United States). In addition, the PSMATCHING3 R Extension command was used (version 2.15.3, R Core Foundation, Austria, Vienna). It was added as an SPSS extension bundle under the SPE file format to run this additional program feature in SPSS.

**Table 1:** Baseline characteristics in the unmatched and matched study populations.

Parameter	Unmatched patients (*n* = 881)			Matched patients (*n* = 336)		
	CABG group	LVAD group	SMD	CABG group	LVAD group	SMD
	*n* = 324	*n* = 557	%	*n* = 168	*n* = 168	%
Age (years)^a^	65.9 ± 10.7	60.0 ± 8.6	61.9	62.6 ± 10.6	62.3 ± 8.0	3.2
Sex, male^b^	282 (87.0)	500 (89.8)	−13.0	148 (88.1)	151 (90.5)	−8.9
Body mass index (kg/m^2^)^a^	27.3 ± 4.7	26.7 ± 4.9	12.5	27.1 ± 5.0	27.0 ± 5.1	2.0
Diabetes mellitus^b^	146 (45.1)	191 (34.3)	31.7	65 (38.7)	61 (36.3)	7.0
Insulin-dependent diabetes mellitus^b^	83 (25.6)	42 (7.5)	91.3	25 (14.9)	26 (15.5)	−2.3
Myocardial infarction^b^	150 (46.3)	106 (19.0)	95.9	58 (34.5)	56 (33.3)	3.5
Mitral regurgitation^a,c^	0.76 ± 0.75	1.20 ± 0.77	−57.9	0.95 ± 0.77	0.99 ± 0.76	−5.2
Cardiac arrest^b^	27 (8.3)	45 (8.1)	1.0	18 (10.7)	19 (11.3)	−2.6
Renal replacement therapy^b^	18 (5.6)	80 (14.4)	−34.3	15 (8.9)	16 (9.5)	−2.8
Chronic obstructive pulmonary disease^b^	66 (20.4)	87 (15.6)	18.1	34 (20.2)	32 (19.0)	4.2
Peripheral vascular disease^b^	62 (19.1)	90 (16.2)	10.8	31 (18.5)	33 (19.6)	−3.8
Stroke^b^	16 (4.9)	63 (11.3)	−27.4	9 (5.4)	9 (5.4)	0.0
Mechanical ventilation^b^	31 (9.6)	99 (17.8)	−29.5	24 (14.3)	21 (12.5)	7.4
Inotropic support^b^	114 (35.2)	429 (77.0)	−139.6	84 (50.0)	92 (54.8)	−13.5
Mechanical circulatory support^2^	42 (13.0)	127 (22.8)	−32.3	32 (19.0)	34 (20.2)	−4.1
MELD-XI^a^	13.3 ± 4.7	15.8 ± 5.6	−48.4	13.8 ± 5.4	13.8 ± 4.6	0.0

CABG, coronary artery bypass grafting; LVAD, left ventricular assist device; SMD, standardized mean difference

MELD-XI, model for end-stage liver disease.

^a^ mean and standard deviation.

^b^ number and percentage.

^c^ scale ranging from 0 to 3.

## 3 Results

### 3.1 Baseline characteristics and PS matching

**Figure 1:**
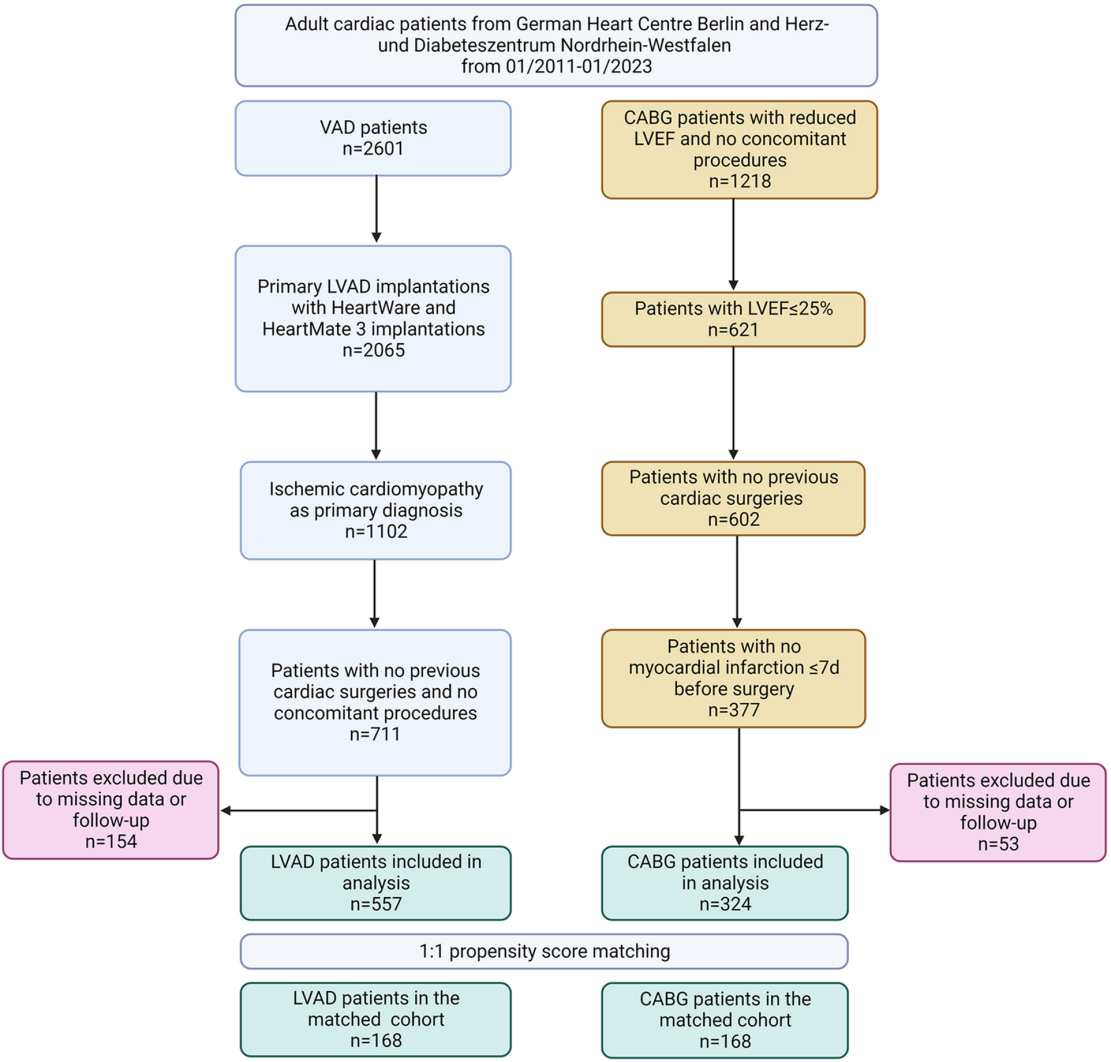
Flow-chart. LVAD, left ventricular assist device; CABG, coronary artery bypass grafting; LVEF, left ventricular ejection fraction.

After excluding patients who did not meet the inclusion criteria and those with missing values, 881 patients were included in the data analysis (Figure 1). In the unmatched groups, the CABG patients were substantially older than the LVAD patients, and presented a higher prevalence of insulin-dependent diabetes mellitus and myocardial infarction (Table 1). In contrast, the incidence of mitral regurgitation, stroke, preoperative RRT, mechanical ventilation, inotropic support, and mechanical circulatory support was substantially higher in the LVAD cohort compared with the CABG group.

In the entire cohort, the PS ranged from a low of 0.000 to a high of 0.998 (Supplementary Figure S1). In the unmatched CABG and LVAD groups, the median (25th to 75th percentiles) PS differed considerably and was 0.318 (0.127–0.547) and 0.869 (0.679–0.947), respectively. PS matching was possible in 38% of the 881 included patients (*n* = 336; Table 1). In the PS-matched CABG and LVAD groups, the PS was 0.540 (0.395–0.742) and 0.561 (0.397–0.771), respectively, and thus very similar. PS matching reduced the standardized differences in preoperative covariates between the study groups substantially (Table 1 and Supplementary Figure S2).

### 3.2 Operative clinical parameters

Off-pump surgery was carried out in 56 (33%) of CABG and in 33 (19.6%) of LVAD patients. The operation time was significantly longer in the CABG group compared with the LVAD group. However, there was no significant difference in cardiopulmonary bypass time between the two study cohorts (Table 2). Mechanical ventilation, ICU stay, and in-hospital stay were significantly longer in LVAD patients. Conversely, secondary clinical endpoints such as the need for RRT and postoperative temporary mechanical support were higher in the CABG group.

**Table 2:** Operative clinical parameters in the matched groups.

Parameter	CABG *n* = 168	LVAD *n* = 168	RR (95%CI)^b^	*P*-value
Operation time, min^a^	209 (184–249)	171 (142–222)	–	<0.001
Cardiopulmonary bypass time, min^a^	76 (0–106)	63 (45–90)	–	0.42
Mechanical ventilation, hours^a^	16 (9–73)	58 (21–256)	–	<0.001
Temporary mechanical support (*n*, %)	51 (30.4)	1 (0.6)	72.8 (9.9–534.2)	<0.001
Intensive care unit stay, days^a^	4 (2–10)	11 (7–24)	–	<0.001
Renal replacement therapy (*n*, %)	15 (8.9)	2 (1.2)	8.1 (1.9–36.2)	0.002
Stroke (*n*, %)	7 (4.2)	11 (6.5)	0.62 (0.23–1.64)	0.47
In-hospital stay (d)	11 (9–17)	28 (20–48)	–	<0.001
In-hospital mortality (*n*, %)	16 (9.5)	24 (14.5)	0.62 (0.32–1.21)	0.18

CABG, coronary artery bypass grafting; LVAD, left ventricular assist device; RR, relative risk; CI, confidence interval; RVAD, right ventricular assist device.

^a^ median with 25th and 75th percentiles.

^b^ reference: LVAD group.

### 3.3 Primary endpoint

**Figure 2:**
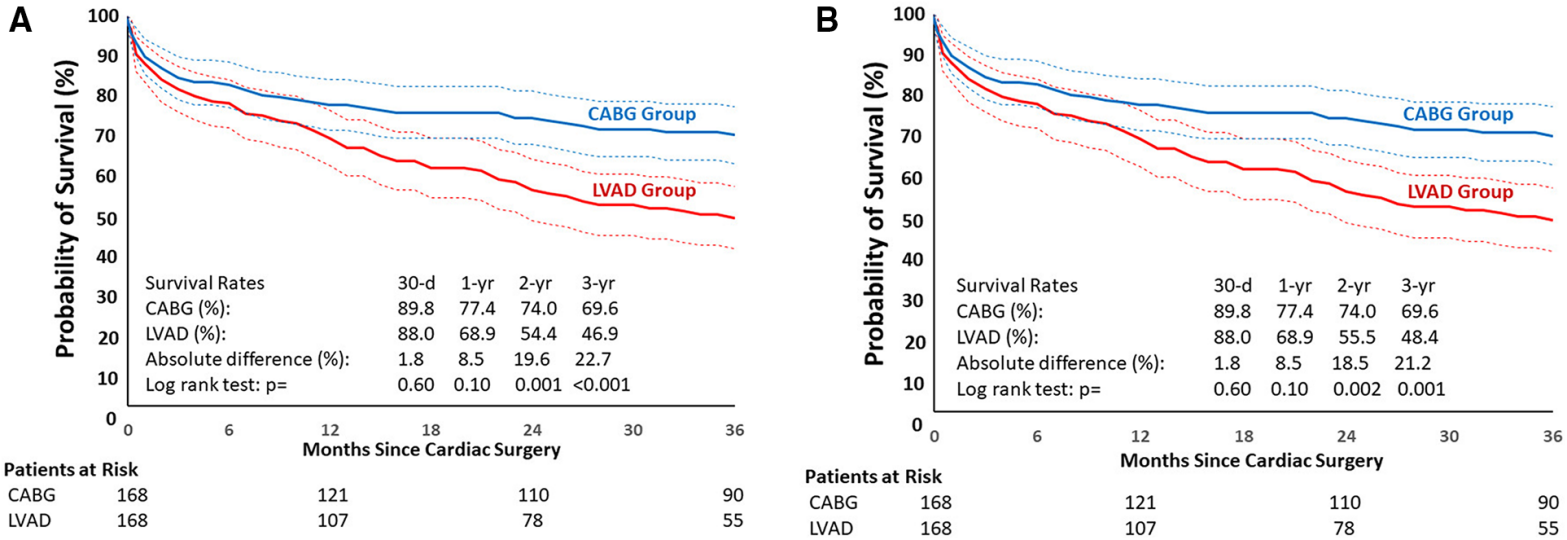
**(A)** Kaplan-Meier estimates in matched cohorts (overall survival). LVAD, left ventricular assist device; CABG, coronary artery bypass grafting. **(B)** Kaplan-Meier estimates in matched cohorts (survival on LVAD support). LVAD, left ventricular assist device; CABG, coronary artery bypass grafting.

An analysis of CABG and LVAD patients after PS-matching showed no differences in 30-day, in-hospital and 1-year survival: 89.8% vs. 88% (*p* = 0.6), 90.5% and 85.5% (*p* = 0.18), and 77.4% vs. 68.9% (*p* = 0.1), respectively. Differences between the groups became significant as of 18 months post-surgery (*p* = 0.013). In particular, the 2- and 3-year survival rates were 74% vs. 54.4% (*p* = 0.001) and 69.6% vs. 46.9% (*p* < 0.001), respectively (Figure 2A).

The corresponding 30-day, 1-year, 2-year, and 3-year HRs (95%CI) of non-survival for the LVAD vs. the CABG group were 1.19 (0.62–2.27), 1.45 (0.95–2.21), 1.86 (1.27–2.72), and 1.95 (1.37–2.78), respectively. The survival rates in matched cohorts were assessed regarding competing events in the LVAD group (LVAD weaning, heart transplantation) demonstrating survival on device (Figure 2B); the results were similar to the overall survival.

In the LVAD group, 6 patients underwent transplantation, 1 was weaned, and another was first weaned and then underwent transplantation during follow-up. Completeness of follow-up at 1 year, 2 years, and 3 years in the CABG and LVAD groups was 93.1 and 93.3%, 88.5 and 85.4%, 60.1 and 50.1%, respectively. In the sensitivity analysis, where all successfully transplanted and weaned patients were considered alive until the last potential follow-up, HRs did not change significantly. In detail, the 30-day value did not change [1.19 (0.62–2.27)]. The 1-year, 2-year, and 3-year values were 1.41 (0.93–2.17), 1.86 (1.52–2.94), and 1.91 (1.34–2.74), respectively.

LVAD-associated complications were recorded throughout the available follow-up period and presented in Table 3 as events per patient-year.

**Table 3:** Complications during follow-up in matched left ventricular assist device patients.

Parameter	Number of patients	Number of events	Events per 100 patient-years
Driveline infection	75	175	52.7
Pump pocket infection	1	1	0.3
Bloodstream infection/sepsis	54	75	22.6
Pump thrombosis	18	44	13.3
Device malfunction	30	64	19.3
Right heart failure	35	44	13.3
Stroke	42	59	17.8
Major bleeding	60	125	37.7
Gastrointestinal bleeding	18	24	7.2

The subgroup analysis for relevant preoperative parameters in unmatched cohorts demonstrated that there was no significant survival difference between CABG and LVAD patients with a history of cardiac arrest, need for inotropic support indicative of cardiogenic shock, or temporary MCS before surgery. Patients with a history of myocardial infarction and ≥70 years of age exhibited a significantly higher mortality risk if they underwent LVAD implantation (Table 4).

**Table 4:** Association between the type of surgery (LVAD or CABG) with a 3-year mortality according to selected preoperative characteristics in unmatched patients.

Parameter	OR (LVAD vs. CABG)	95% CI	*P*-value
Age ≥ 70 years of age	3.95	(2.29–6.80)	<0.001
Myocardial infarction	2.54	(1.52–4.27)	<0.001
Cardiac arrest	2.72	(0.79–9.36)	0.11
Stroke	0.82	(0.29–2.34)	0.71
Preoperative inotropic support	1.42	(0.79–2.54)	0.24
Temporary mechanical circulatory support	1.79	(0.65–4.93)	0.26

## 4 Discussion

### 4.1 Interpretation of the results

To the best of our knowledge, the presented study is the first PS-matched analysis comparing the results of CABG vs. LVAD implantation in patients with CAD, advanced HF and a severely reduced LVEF (≤25%) in terms of mid-term survival. In this analysis, we found no survival difference up to 18 months post-surgery in CAD patients with an LVEF ≤25% undergoing LVAD implantation or CABG. Beyond the 18-month follow-up, CABG patients have a significantly better survival compared to the LVAD cohort. The survival curves in matched cohorts diverged right from the beginning; however, the significance threshold was only reached after 18 months of follow-up.

One possible explanation for the higher mid-term mortality in LVAD group could be the impact of LVAD-associated complications over time. Durable LVAD represents a feasible option in patients with end-stage HF; however, existing devices are associated with a continuously decreasing but ongoing burden of complications (11).

LVAD-associated infections are the major cause of hospital admission among LVAD patients, occurring in 30%–40% of cases in the first follow-up year (12). The main source of LVAD-associated infections remains the driveline which penetrates the skin, disturbing the natural barrier and rendering the exit site susceptible to chronic colonization by pathogens and biofilm-building bacteria (13). In our population, 44.6% of patients were diagnosed with DL-infections with a total of 175 events during the follow-up period. However, it should be noted, that the rate of infections was observed throughout the whole follow-up period, exceeding the 1-year benchmark in some cases.

Mandatory postoperative anticoagulation with vitamin K antagonists is crucial for mitigating thromboembolic complications associated with LVAD use, albeit at the cost of an elevated bleeding risk (11, 14). Stroke incidents, encompassing both thromboembolic and hemorrhagic cases, transpired in 25% of our LVAD patients, underlining the pivotal role of deranged anticoagulation in contributing to neurological events (14).

### 4.2 Patient selection

In our study, PS matching was possible only in 38% of all patients enrolled in the analysis, indicating a significant difference in patient populations, which plays a major role in the decision process. A comparison of patients excluded by matching (CABG *n* = 156 and LVAD *n* = 389) revealed that CABG patients were older, had a higher prevalence of diabetes, and a higher incidence of previous myocardial infarction at baseline (Table 5). At the same time, the LVAD group was more likely to be on invasive ventilation, preoperative inotropes, or temporary mechanical circulatory support.

**Table 5:** Baseline characteristics in excluded patients.

Parameter	CABG group *n* = 156	LVAD group *n* = 389	*p*-value
Age (years)^a^	69.4 ± 9.6	58.9 ± 8.6	<0.001
Sex, male^b^	134 (85.9)	348 (89.5)	0.24
Body mass index (kg/m^2^)^a^	27.5 ± 4.4	26.6 ± 4.9	0.034
Diabetes mellitus^b^	81 (51.9)	130 (33.4)	<0.001
Insulin-dependent diabetes mellitus^b^	58 (37.2)	16 (4.1)	<0.001
Major myocardial infarction^b^	92 (59.0)	50 (12.9)	<0.001
Mitral regurgitation^c,d^	0 (0–1)	1 (1–2)	<0.001
Cardiac arrest^b^	9 (5.8)	26 (6.7)	0.85
Renal replacement therapy^b^	3 (1.9)	64 (16.5)	<0.001
Chronic obstructive pulmonary disease^b^	32 (20.5)	55 (14.1)	0.07
Peripheral vascular disease^b^	31 (19.9)	57 (14.7)	0.16
Stroke^b^	7 (4.5)	54 (13.9)	0.001
Mechanical ventilation^b^	7 (4.5)	201 (20.1)	<0.001
Inotropic support^b^	30 (19.2)	93 (23.9)	<0.001
Mechanical circulatory support^b^	10 (6.4)	127 (22.8)	<0.001
MELD-XI^c^	12.0 (9.4–14.2)	15.8 (11.7–20.7)	<0.001

CABG, coronary artery bypass grafting; LVAD, left ventricular assist device; MELD-XI, model for end-stage liver disease.

^a^ mean and standard deviation.

^b^ number and percentage.

^c^ median with 25th and 75th percentiles.

^d^ scale ranging from 0 to 3; abbreviations.

The subgroup analysis of unmatched cohorts demonstrated that patients with a history of myocardial infarction and ≥70 years of age had a significantly better survival if they underwent CABG. This finding advocates for a revascularization strategy in CAD patients with a reduced LVEF, especially since bypass grafting might potentially reduce the risk of future infarctions after the surgery. Surprisingly, the ORs for inotropic support and preoperative MCS were not significant. This finding must be interpreted with caution, since the LVAD cohort had a higher prevalence of patients requiring circulatory support in unmatched cohorts. Additionally, based on our data we cannot stratify groups by the level of support required prior to surgery. In this constellation, the question whether to perform CABG or LVAD implantation in hemodynamically compromised patients remains open.

It should be underlined that, even though the above-mentioned parameters play a role in the preoperative therapy decision, the analysis was performed in unmatched cohorts, so that confounding factors should be taken into consideration.

### 4.3 CABG vs. medical therapy

Modern medical therapy has undoubtedly made substantial advancements in improving morbidity and mortality among HF patients (2, 3). However, in the case of ischemic HF patients, surgical revascularization proves to be superior to medical therapy alone (15). The landmark STICH (Surgical Treatment for Ischemic Heart Failure) trial has demonstrated a significant reduction in mortality and HF-related hospitalizations over a follow-up period of up to 10 years (15). It is worth emphasizing that even after surgery, patients still require guideline directed HF medication for optimal management (4, 15).

### 4.4 PCI in HF patients with reduced LVEF

Percutaneous coronary intervention (PCI) is often considered a less invasive alternative for revascularization in patients with ischemic HF, particularly in high-risk cases (16). The Study of Efficacy and Safety of Percutaneous Coronary Intervention to Improve Survival in Heart Failure (REVIVED-BCIS2) is the first prospective randomized trial to investigate whether PCI revascularization can improve outcomes in patients with reduced LVEF and CAD compared to the guideline directed HF medication alone (16). However, the REVIVED-BCIS2 trial did not demonstrate a significant benefit or reduction in hospitalization rates for patients undergoing PCI. After a two-year follow-up, the all-cause mortality rate was 31.7% for the PCI cohort and 32.6% for the medical therapy cohort (16). The prevalence of acute myocardial infarction (AMI) remained similar between the groups, with rates of 10.7% and 10.8% respectively (16). However, it is important to underline, that almost 40% of AMI in PCI cohort occurred as periprocedural complications (16).

In observational studies, CABG appears superior to PCI in patients with ischemic left ventricular dysfunction (5). Sun et al. showed in a matched cohort of 4,794 patients that PCI was associated with higher risk of 5-year mortality (30.0% PCI group vs. 23.3% CABG group) and death from cardiovascular disease (3.5% vs. 2.8%), MACE (19.8% vs. 8.3%), subsequent revascularization (10.9% vs. 3.2%), and hospitalization for MI (7.8% vs. 1.4%) and HF (5.6% vs. 3.0%) compared with CABG in patients with CAD and LVEF less than 35% (5). However, large randomized controlled studies are lacking in the literature.

### 4.5 Myocardial viability

Acute or chronic ischemia on top of CAD is the leading mechanism causing loss of myocardial contractility (2). The resulting systolic dysfunction is caused not only by irreversible myocardial fibrosis but also by a reversible dysfunction of vital cardiomyocytes (17). So-called myocardial stunning (hibernation) is described as prolonged post-ischemic dysfunction of vital cells, which is potentially reversible after restoring adequate blood perfusion (17). Modern imaging techniques such as contrast-enhanced magnet resonance imaging (MRI) and positron emission tomography (PET) enable the visualization of myocardial viability through the uptake and retention of metabolically active tracers (18). Preoperative evaluation of myocardial viability is a promising approach for an indication for revascularization. However, scientific evidence on the effectiveness of revascularization of hibernated myocardium remains limited (17).

At our centers, myocardial viability imaging serves as an important decision-making support tool in CAD patients with HF. However, it is not performed routinely in every patient. Since no clear evidence for viability testing was presented in large randomized trials so far cardiac MRI was not performed routinely in all patients (19, 20). A recently published substudy of the REVIVED-BCIS2 trial demonstrated no difference in primary outcomes between patients who underwent complete anatomical and viability-guided revascularization and those who received medical therapy for heart failure or incomplete revascularization (21). Additionally, the use of functional imaging, especially in inotropic-dependent patients or patients on temporary MCS is not standardized. Due to retrospective nature of our study we could not provide data on myocardial viability and could not follow if the decision whether to perform an LVAD implantation or CABG was influenced by the results of viability assessment.

## 5 Limitations

In our study, like in other bicentric studies, we encountered missing data. To ensure data completeness and accuracy, we implemented various measures such as data input control, on-site audits, and statistical analyses to enhance data quality.

Both participating centers are highly experienced, high-volume hospitals that specialize in treating end-stage heart failure (HF) patients. The decision whether to perform CABG or LVAD in each individual case was made through discussions among a multidisciplinary heart team. Important criteria for therapy decision-making included the coronary status of the patient as well as the myocardial viability or scar tissue extension. Unfortunately, due to the retrospective nature of presented study, we cannot provide data on myocardial viability, coronary targets and structured decision-making protocols of the heart team in order to highlight the indication for one or another treatment in each case. However, it is important to note that, despite PS-matching for viability, extent of CAD and other potential confounders, a selection bias cannot be excluded due to the observational character of our study.

The surgical strategy during the CABG (with or without cardioplegic heart arrest, with or without CPB establishment) as well as the device type for LVAD implantation (HW or HM3) play a significant role on the outcomes of patients. Future studies with a certain surgical technique should be conducted. In our case, limited number of patients precluded us from performing such analysis.

A statistical power calculation revealed that the sample size had to be 446 (223 patients in each group) to detect a relative difference of 45% in the primary endpoint at the 12-month follow-up in the LVAD group compared with the CABG group. This calculation is based on a statistical power of 90 percent, a two-sided 5% significance level, and the assumption that the accrual period is 150 months, and the median survival is 36 months.

Another issue we encountered was a notably lower rate of late follow-up data in the CABG group compared to the LVAD group. LVAD patients participate in regular outpatient check-ups and if follow-up complications occur, the treatment takes place in the respective cardiac center or in close cooperation with it, resulting in more data. In case of CABG group, no systematic check-up program exists. In case of patients’ death, the date of death could be obtained via local authorities, but the reason of death was missing.

However, it is worth mentioning that more deaths occurred in the LVAD group. Cohorts with a higher early event rate may naturally have a lower follow-up rate due to a smaller risk set (22).

Ultimately, to answer questions as to the specific advantages of CABG or LVAD implantation for each individual patient, a multicenter, prospective, randomized study is necessary.

## 6 Conclusion

In patients with CAD and a reduced LVEF, there was no significant difference in survival between those who underwent either CABG or LVAD implantation up to 18 months post-surgery. However, beyond this time period, the CABG group demonstrated significantly better survival rates. These findings add valuable insights to the ongoing debate regarding the optimal treatment approach for patients with advanced ischemic HF. Prospective randomized studies comparing surgical revascularization with durable LVAD implantation in patients with CAD and reduced LVEF are highly warranted.

## Data Availability

The data analyzed in the study was partially provided by the EUROMACS database of the EACTS and cannot be provided to a third party without permission by the EUROMACS. Requests to access the datasets should be directed to the corresponding author.

## Supplementary material

Supplementary Figure S1 Standardized difference before and after matching.

Supplementary Figure S2 Distribution of propensity scores.
