# Supplementary figures and images for: Propensity score-based comparison of high-risk coronary artery bypass grafting vs. left ventricular assist device implantation in patients with coronary artery disease and advanced heart failure
Source: Front Cardiovasc Med. 2024 Oct 1;11:1430560. doi: 10.3389/fcvm.2024.1430560 (PMC11473413; doi:10.3389/fcvm.2024.1430560)

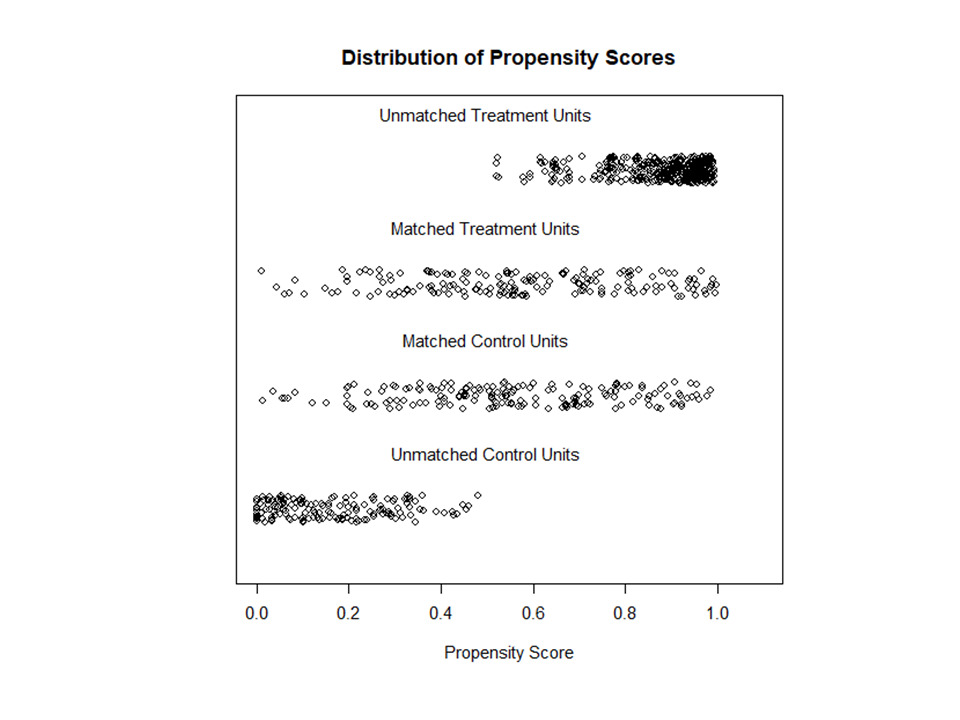

Supplement: Supplementary Figure S1 [file Image1.jpeg]

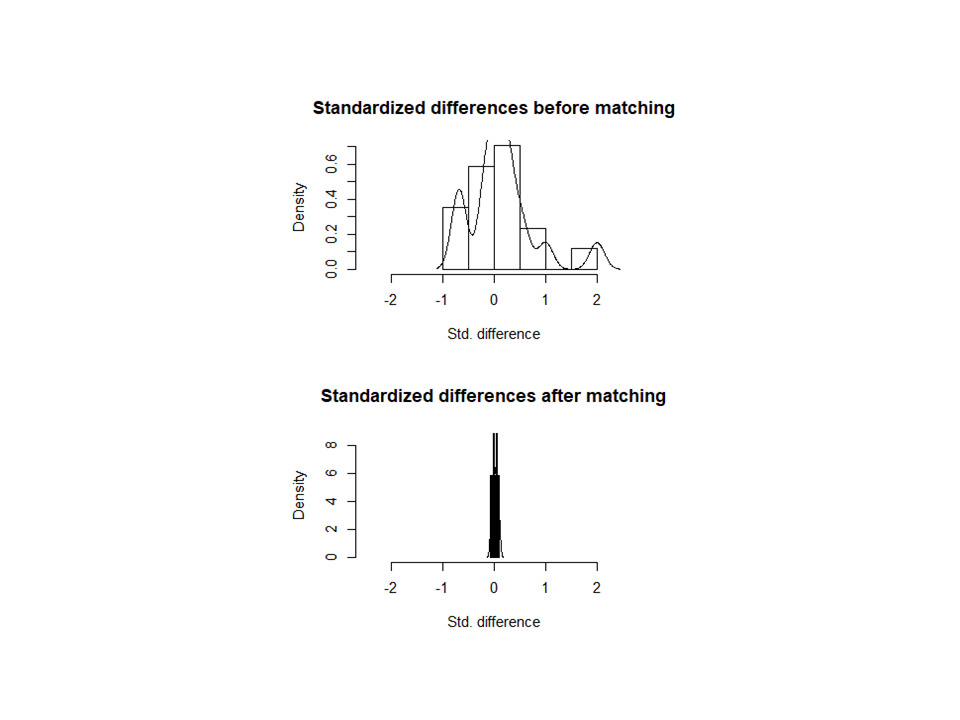

Supplement: Supplementary Figure S2 [file Image2.jpeg]
